# The evolution of the impact of educational background on oral health: A bibliometric and visualized analysis from 2000 to 2024

**DOI:** 10.1097/MD.0000000000043795

**Published:** 2025-08-08

**Authors:** Linxin Jiang, Bincheng Liu, Simin Li, Shaohong Huang, Daniel R. Reissmann, Gerhard Schmalz, Xianda Hu

**Affiliations:** a Department of Prosthodontics, University of Leipzig, Leipzig, Germany; b Stomatological Hospital, School of Stomatology, Southern Medical University, Guangzhou, Guangdong, China; c Liwan District Stomatological Hospital, Guangzhou, Guangdong, China; d Department of Conservative Dentistry and Periodontology, Brandenburg Medical School Theodor Fontane (MHB), Brandenburg/Havel, Germany; e Laboratory of Molecular Cell Biology, Beijing Tibetan Hospital, China Tibetology Research Center, Chaoyang, Beijing, China.

**Keywords:** bibliometric analysis, education, oral health, oral health disparities, socio-economic factors

## Abstract

**Background:**

Educational background is a major associated factor on oral health. However, comprehensive analyses of global research trends in this field remain limited. This study aimed to analyze the current status, hotspots, and developmental trends in research on the impact of educational background on oral health.

**Methods:**

This study conducted a comprehensive bibliometric analysis using data from the Web of Science Core Collection database, covering the period between 2000 and 2024. The search strategy combined terms related to education and oral health. A total of 1080 articles were retrieved and analyzed using GraphPad 8.0.2, CiteSpace 6.2. R6, and VOSviewer 1.6.20 visualization software. This study examined publication trends, countries/regions, journals, institutions, authors and research hotspots.

**Results:**

The analysis revealed a steady increase in publications over the study period, with a notable surge in the past 4 years. The United States of America, the United Kingdom, Australia, China, and Brazil emerged as the leading countries. With the exception of 1 institution and 1 author from Brazil, the top 5 institutions, authors, and journals in terms of publication, citation, and collaboration strength are all from developed countries. Keyword co-occurrence analysis revealed that the predominant research keywords are centered most around health literacy. Keywords clustering grouped the terms into twelve categories, and the cluster of oral health education is the most highly focused area. The analysis of keyword temporal trends reveals a gradual decline in prominent research clusters over time, from 7 to 5 and then to 4. From 2020 to 2024, oral health knowledge, older adults, diseases, and students are emerging hotspots.

**Conclusion:**

The research landscape on the impact of education on oral health is dynamic and holds significant potential for further development. Enhanced collaboration among countries, institutions, and authors is essential to foster interdisciplinary advancements and address global challenges within this field. Recent trends in research indicate a growing specialization within subdomains and an increasing emphasis on early prevention.

## 1. Introduction

Oral health is an essential component of overall health and quality of life,^[1]^ with educational background being a key social determinant that profoundly influences oral health status.^[2,3]^ Over the past few decades, numerous studies have explored the complex relationship between educational background and oral health. Studies consistently demonstrate that higher educational levels are associated with better oral health knowledge and practices, which can lead to improved oral health outcomes.^[4–7]^ This relationship is evident across various populations. Research indicated that among young adults, higher education levels correlated with better oral hygiene practices, including regular use of toothbrushes and interdental cleaners, while lower educational levels were associated with harmful practices like smoking and chewing Arabic tea.^[8]^ The children with higher parental education levels generally exhibit more oral health knowledge, better oral hygiene habits, and more frequent dental visits.^[6,9,10]^ Elderly with lower education levels tend to have poorer oral health-related quality of life.^[11]^ However, the elderly individuals cared for by healthcare workers who have received oral health education show a significant increase in the proportion of those with normal oral mucosa, no visible dental plaque, and no denture-related stomatitis.^[12]^ Despite the abundance of literature, systematic reviews and analyses of overall trends and key hotspots in this field remain insufficient, particularly with respect to global comparative studies and long-term trend analyses. Furthermore, recent developments such as the accelerating global aging population^[13]^ and the disruptive impact of the COVID-19 pandemic^[14]^ have introduced potentially significant but largely unexplored influences on this domain. These gaps underscore the substantial need for further research in these emerging areas.

Bibliometrics, a quantitative method for analyzing literature characteristics, offers a macroscopic perspective on the developmental trajectory, hot topics, and future trends in specific research fields.^[15]^ This method, pioneered by Price and Garfield in the 1960s, has since been widely applied across various disciplines. Through objective and systematic analysis of large volumes of literature data, bibliometrics aids researchers in identifying key authors, institutions, and journals, understanding the evolution of research themes, and uncovering potential research gaps. Previous studies have successfully utilized bibliometric analysis to investigate the impact of various factors on oral health, such as diet,^[16]^ psychological stress,^[17]^ and systemic diseases (e.g., rheumatoid arthritis,^[18]^ diabetes.^[19]^ For the complex and broad field of “the impact of educational background on oral health,” employing bibliometric analysis presents unique advantages.

This study aims to systematically analyze the current status, hotspots, and developmental trends in research on the impact of educational background on oral health using bibliometric methods. By analyzing indicators such as publication volume, citation frequency, countries/regions distribution, institutional collaboration networks, and high-frequency keywords, this study will uncover research frontiers, major contributors and their collaborative relationships, as well as the evolution of research themes in this area. This multi-dimensional analysis seeks to bridge existing knowledge gaps, offering a clearer understanding of development trends in the field while providing valuable insights to inform future research and strategies for addressing oral health disparities. Based on the existing research and unexplored factors, this study proposed the following hypotheses: The volume of research in this field had significant fluctuations in the recent years, with notable variations in the volume and impact of research across different countries and regions; greater attention to specific populations and more refined subfields are emerging directions.

## 2. Materials and methods

### 2.1. Data source

Considering that Web of Science (WoS) is one of the world’s most important academic databases and is frequently used for bibliometric analysis, the WoS Core Collection database was selected as the source of literature. This study queried the Science Citation Index Expanded edition of the WoS Core Collection database.

The search strategy retrieved studies with education-related titles and oral health-related topics, while excluding those with titles that fall outside the scope of this study. After careful consideration and refinement of related terms, search strategy was finalized as follows:

TI = (Education Level OR Educational Attainment OR Education OR Literacy OR Educational inequalities) AND TS = (caries OR dental decay OR tooth decay OR denture* OR oral hygiene OR dental plaque OR oral health OR tooth loss OR dental prosthesis* OR periodont* OR dry mouth OR oral dryness OR xerostomia OR toothache* OR edentulism OR oral pain OR number of teeth OR dentin sensitivity OR tooth sensitivity OR oral ulcer* OR mouth ulcer* OR gingivitis OR gingival bleeding OR oral cancer* OR oropharyngeal cancer* OR oral dysbiosis OR oral frailty OR oral hypofunction OR dental pain OR endodontic disease OR periodontitis OR periodontal disease OR oral squamous cell carcinoma OR oral mucosal lesions OR loss of tooth OR awareness) NOT TI = (dental education OR medical education OR nurse practitioner education OR nurs* education OR medical student* OR dental student* OR professionals OR strateg* OR train* OR examination* OR course* OR curriculum* OR dental examination OR dental faculty OR dental school* OR dental education program OR dental program OR dental hygienists OR dentist* OR interprofessional education)

Data collection was performed on December 3, 2024. The Inclusion Criteria were: Articles and Reviews, English language, and publications between 2000 and 2024. The Exclusion Criteria included: conference proceedings, early access publications, and editorial materials. This process yielded a total of 1080 articles, which were exported in plain text format for record-keeping and citation purposes.

### 2.2. Analysis tools

For this study, 3 visualization analysis software tools were employed: GraphPad Prism 8.0.2 (GraphPad Software, San Diego), CiteSpace 6.2.R6 (Chaomei Chen, Drexel University, Philadelphia),^[20]^ and VOSviewer 1.6.20 (Centre for Science and Technology Studies, Leiden University, Leiden, The Netherlands).^[21]^ The combined use will allow this study to explore the hot topics, trends, and evolution of research related to the impact of educational background on oral health more thoroughly.

GraphPad visualizes annual publishing trends. CiteSpace enables the clustering of keywords, the visualization of keyword evolution through a timeline view, and the detection of keyword bursts. VOSviewer can construct collaborative networks based on countries/regions, institutions, authors, and journals, as well as co-occurrence network based on keywords.

**Figure 1.**
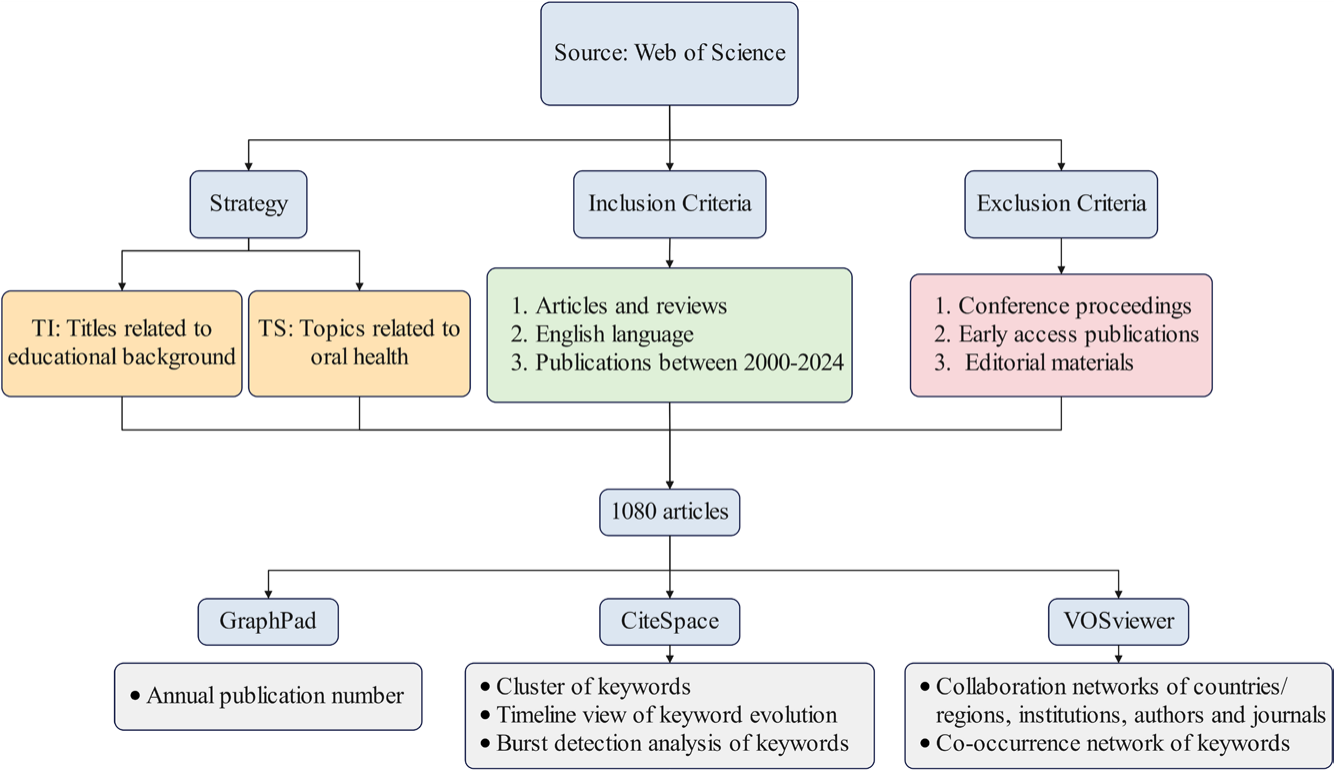
Flow diagram of the study.

Figure 1 shows analytic approach and study flow.

## 3. Results

### 3.1. Annual publication trend

**Figure 2.**
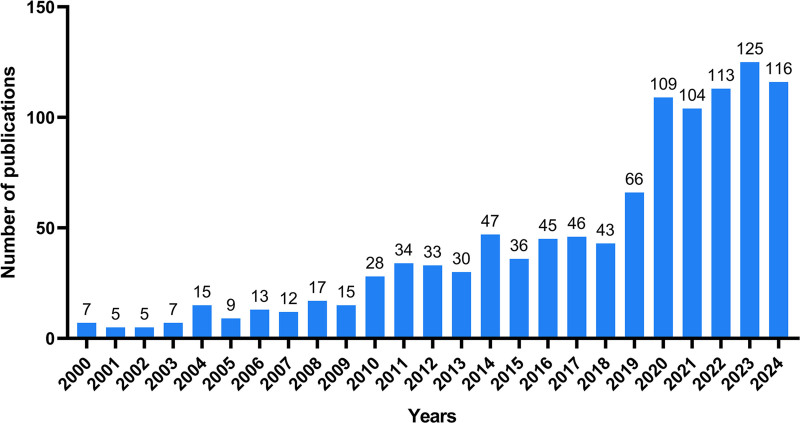
Annual publication trend on the educational impact on oral health (2000–2024).

Figure 2 shows the publication trend of literature on “the impact of educational background on oral health” from 2000 to 2024. In the early phase (2000–2009), the average was around 10 articles per year, for a total of 105 publications. In the mid-phase (2010–2019), the annual number of publications increased from 28 in 2006 to 66 in 2019. In recent years (2020–2024), the number of publications increased from 109 in 2020 to 116 in 2023. Data collection for 2024 is still in progress and has reached 112.

### 3.2. Countries/Regions analysis

Table 1 demonstrates that the USA ranks first in the number of publications, with 340 articles, surpassing the combined total of the next 4 countries. Following the USA are Australia, Brazil, the UK, China, India, Iran, Canada, Germany, Saudi Arabia. The USA also leads in citation frequency with 12,394 citations, exceeding the combined total of the second to tenth-ranking countries. The subsequent countries in terms of citations are the UK, Australia, China, Brazil, Canada, Netherlands, Denmark, Germany, Iran.

**Table 1:** The top 10 countries/regions ranked by the number of publications, citations, and collaboration link strength in studies on the educational impact on oral health.

Rank	Country/region	Publications	Country/region	Citations	Country/region	Total link strength
1	USA	340	USA	12,394	USA	177
2	Australia	96	UK	2218	UK	132
3	Brazil	89	Australia	1733	Brazil	117
4	UK	71	China	1493	China	111
5	China	66	Brazil	1352	Australia	108
6	India	61	Canada	932	Canada	97
7	Iran	50	Netherlands	890	Norway	87
8	Canada	45	Denmark	686	Germany	86
9	Germany	38	Germany	624	Switzerland	86
10	Saudi Arabia	34	Iran	594	France	80

**Figure 3.**
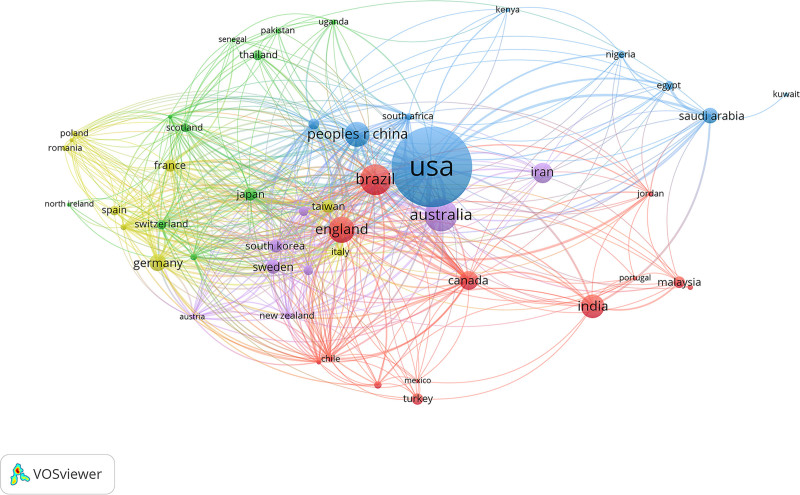
The collaboration network of countries/regions in studies on the impact of educational background on oral health from 2000 to 2024.

For the collaboration analysis (Fig. 3), link strength reflects the intensity of research collaboration and academic networks between countries or regions. The USA exhibits the highest link strength, followed by the UK, Brazil, China, Australia, Canada, Norway, Germany, Switzerland, France.

### 3.3. Institution analysis

**Figure 4.**
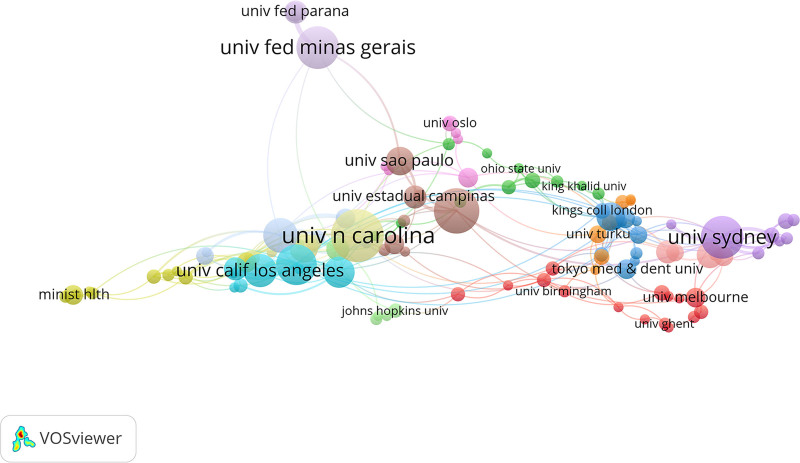
The collaboration network of institutions in studies on the impact of educational background on oral health from 2000 to 2024.

Table 2 and Figure 4 reveal that the University of North Carolina not only leads in volume (30 articles) but also enjoys widespread academic recognition and influence, with a total of 4286 citations. Additionally, the link strength ranks seventh (18). University of Adelaide ahd Universidade Federal de Minas Gerais rank second and third in terms of publication volume, while Duke University and RTI International occupy the second and third positions in citation frequency. In terms of link strength, the top 3 are University of Maryland, Harvard University, and University of Sydney.

**Table 2:** The top 10 institutions ranked by the number of publications, citations, and collaboration link strength in studies on the educational impact on oral health.

Rank	Institutions	Publications	Institution	Citations	Institution	Total link strength
1	University of North Carolina	30	University of North Carolina	4286	University of Maryland	31
2	University of Adelaide	25	Duke University	3306	Harvard University	26
3	Universidade Federal de Minas Gerais	23	RTI International	3304	University of Sydney	25
4	University of Sydney	23	University of California, San Francisco	995	Universidade Federal de Minas Gerais	21
5	University Maryland	22	Emory University	872	Northwestern University	19
6	Harvard University	18	Harvard University	734	University of California, Los Angeles	19
7	University of California, Los Angeles	17	University of Maryland	717	University of North Carolina	18
8	University of California, San Francisco	16	University of Hong Kong	707	Emory University	17
9	University of Hong Kong	14	Boston University	555	University of Adelaide	16
10	University of Michigan	14	University of Michigan	551	RAND Corporation	16

### 3.4. Author analysis

**Figure 5.**
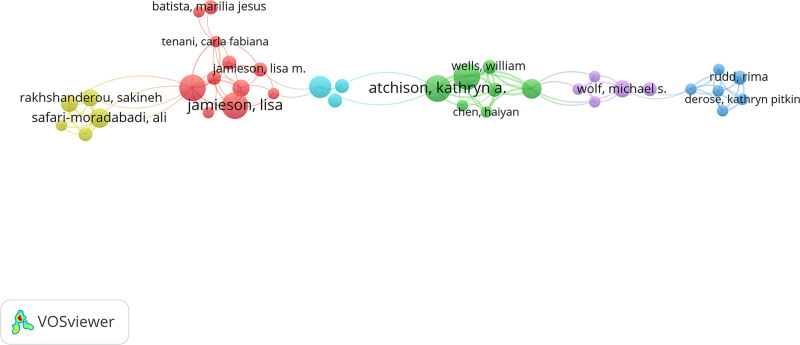
The collaboration network of authors in studies on the impact of educational background on oral health from 2000 to 2024.

Table 3 and Figure 5 illustrate that the top 3 authors in terms of publication volume are “Atchison, Kathryn A.,” “Jamieson, Lisa,” “Macek, Mark D..” “Atchison, Kathryn A.,” “Macek, Mark D.” also demonstrate strong collaborative networks, ranking second and third in link strength. Regarding citation frequency, the top 3 authors are “Wolf, Michael S.,” “Parker, Ruth M.,” “Dreyer, Benard P..” And “Parker, Ruth M.” has the highest link strength.

**Table 3:** The top 10 authors ranked by the number of publications, citations, and collaboration link strength in studies on the educational impact on oral health.

Rank	Authors	Publications	Author	Citations	Author	Total link strength
1	Atchison, Kathryn A.	9	Wolf, Michael S.	367	Parker, Ruth M.	27
2	Jamieson, Lisa	9	Parker, Ruth M.	359	Atchison, Kathryn A.	22
3	Macek, Mark D.	9	Dreyer, Benard P.	242	Macek, Mark D.	22
4	Mialhe, Fabio Luiz	9	Mendelsohn, Alan L.	242	Mialhe, Fabio Luiz	22
5	Horowitz, Alice M.	7	Yin, H. Shonna	242	Haynes, Don	19
6	Parker, Ruth M.	6	Atchison, Kathryn A.	232	Wells, William	19
7	Safari-Moradabadi, Ali	6	Macek, Mark D.	208	Safari-Moradabadi, Ali	18
8	Ghaffari, Mohtasham	5	Horowitz, Alice M.	166	Ghaffari, Mohtasham	18
9	Ju, Xiangqun	5	Haynes, Don	157	Rakhshanderou, Sakineh	18
10	Rakhshanderou, Sakineh	5	Wells, William	157	Chen, Haiyan	16

### 3.5. Journal analysis

**Figure 6.**
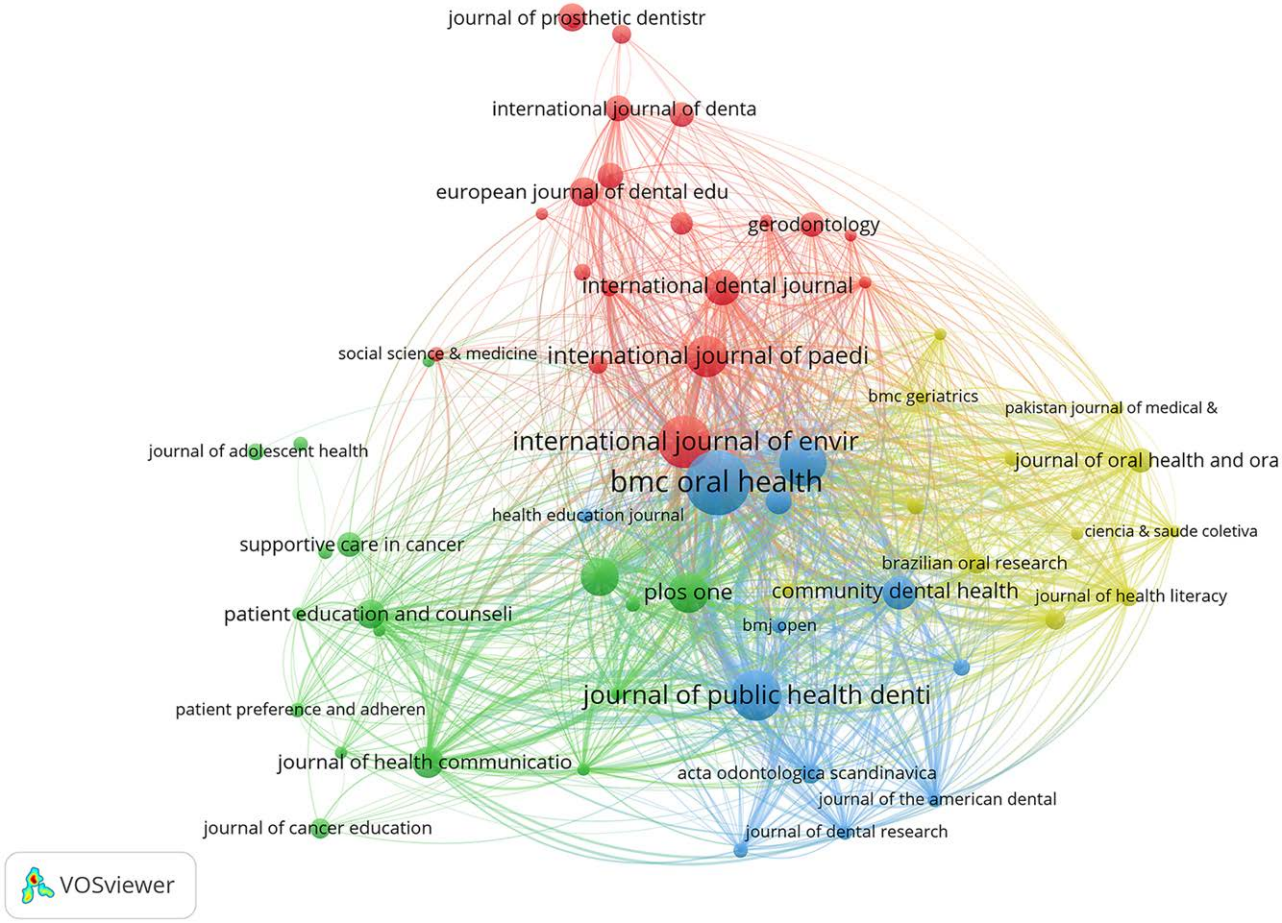
The collaboration network of journals in studies on the impact of educational background on oral health from 2000 to 2024.

Table 4 and Figure 6 reveal that the BMC Oral Health holds the top position in both publication volume, citation frequency, and link strength. Journals that place in the top 5 across all 3 metrics include the Journal of Public Health Dentistry and Community Dentistry and Oral Epidemiology.

**Table 4:** The top 10 journals ranked by the number of publications, citations, and collaboration link strength in studies on the educational impact on oral health.

Rank	Journal	Publications	Journal	Citations	Journal	Total link strength
1	BMC Oral Health	39	BMC Oral Health	846	BMC Oral Health	7983
2	Int J Environ Res Public Health	29	Community Dent Oral Epidemiol	731	J Public Health Dent	5738
3	J Public Health Dent	28	Cochrane Database Syst Rev	718	Community Dent Oral Epidemiol	5357
4	Community Dent Oral Epidemiol	25	Patient Educ Couns	688	Plos One	3770
5	Int J Paediatr Dent	21	J Public Health Dent	631	Int J Environ Res Public Health	3765
6	Plos One	20	Int J Paediatr Dent	628	BMC Public Health	3062
7	BMC Public Health	19	J Health Commun	463	Community Dent Health	2983
8	Int Dent J	17	BMC Public Health	407	J Health Commun	2374
9	Community Dent Health	16	J Clin Periodontol	392	Int J Paediatr Dent	2373
10	J Health Commun	14	Plos One	386	J Health Lit	2357

### 3.6. Keyword co-occurrence analysis

**Figure 7.**
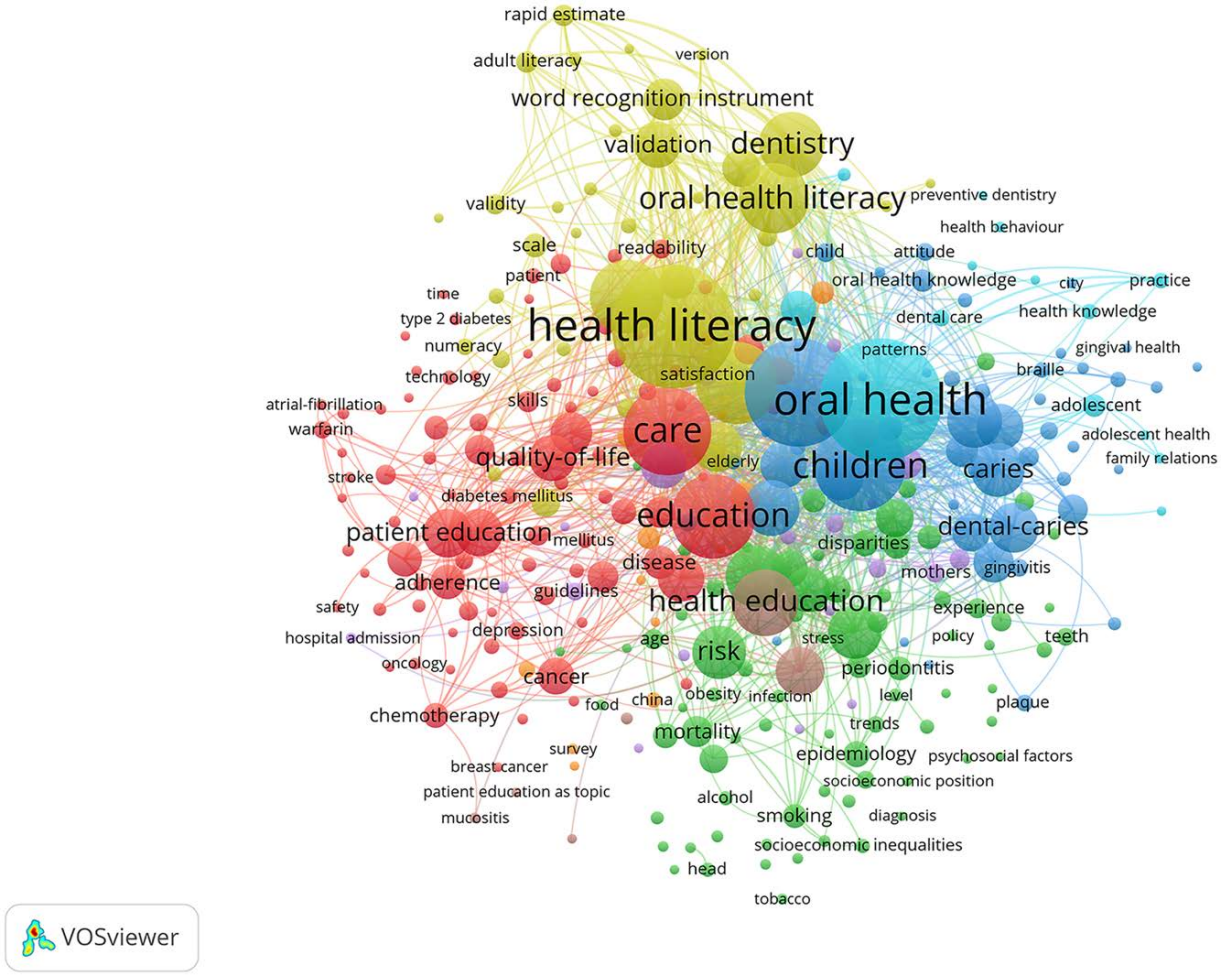
The co-occurrence network of keywords in studies on the impact of educational background on oral health from 2000 to 2024.

The top 3 keywords associated with occurrence and link strength all include health literacy, oral health, and knowledge (Fig. 7).

### 3.7. Keyword clustering analysis

**Figure 8.**
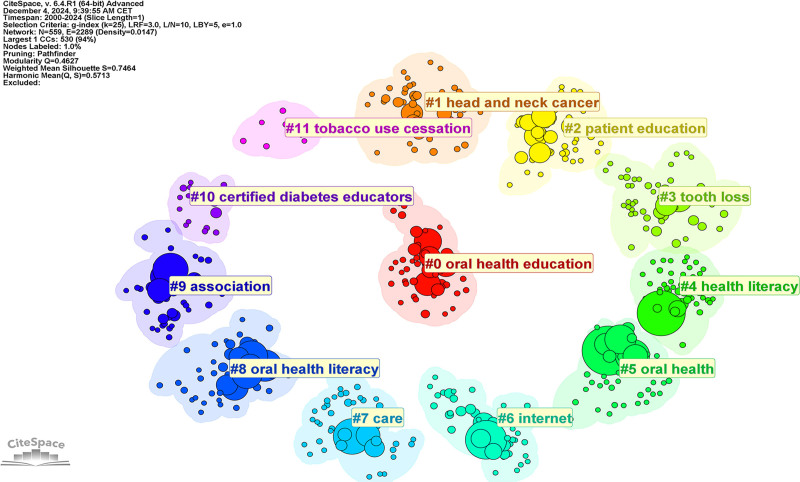
The cluster of keywords in studies on the impact of educational background on oral health from 2000 to 2024.

The CiteSpace keyword clustering knowledge map identified twelve clusters, including oral health education, head and neck cancer, patient education, tooth loss, health literacy, oral health, internet, care, oral health literacy, association, certified diabetes educators, tobacco use cessation. Number of publications or influence in each cluster are inversely proportional to the cluster id (Fig. 8). The main keywords included in each cluster can be found in Table S1, Supplemental Digital Content, https://links.lww.com/MD/P613. The network consists of 559 nodes and 2289 connections, with a density of 0.0147. The modularity score of 0.4627 indicates a reasonable modular structure, reflecting the thematic distribution within the research domain. Additionally, the weighted mean silhouette of 0.7464 suggests a high level of separation and internal consistency among the clusters, collectively demonstrating the credibility of the clustering results.

### 3.8. Keyword temporal trend analysis

**Figure 9.**
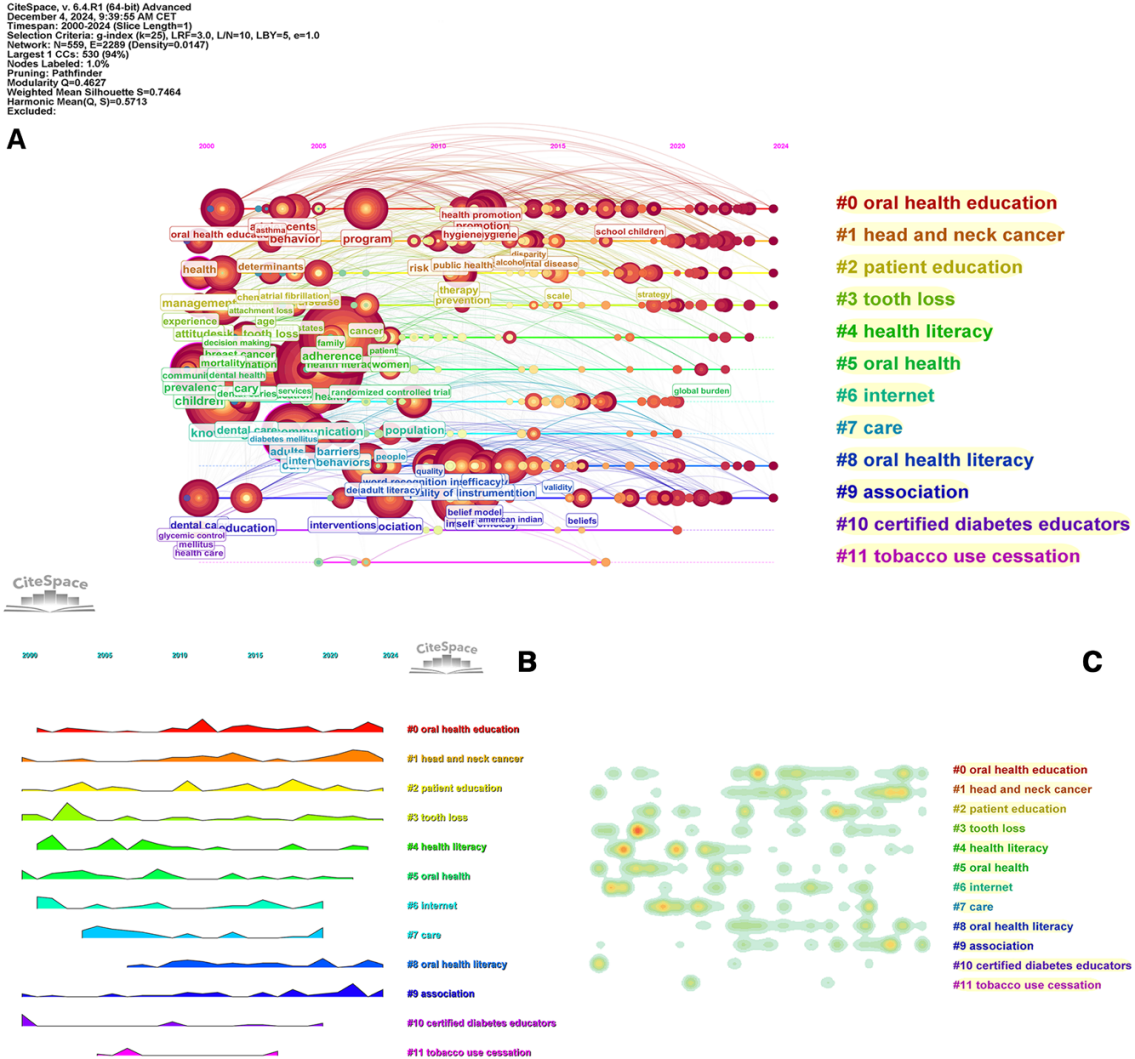
Keyword evolution in studies on the impact of educational background on oral health from 2000 to 2024. (A) Timeline view; (B) landscape view; (C) heatmap view.

The CiteSpace keyword timeline analysis network encompasses 12 clusters (Fig. 9). During the early phase (2000–2009), the most prominent keyword clusters were “patient education,” “tooth loss,” “health literacy,” “oral health,” “internet,” “care,” and “certified diabetes educators.” From 2010 to 2019, the focus shifted to “oral health education,” “head and neck cancer,” “patient education,” “internet,” and “oral health literacy.” In the most recent period (2020–2024), attention has further concentrated on “oral health education,” “head and neck cancer,” “oral health literacy,” and “association.”

### 3.9. Keyword burst analysis

**Figure 10.**
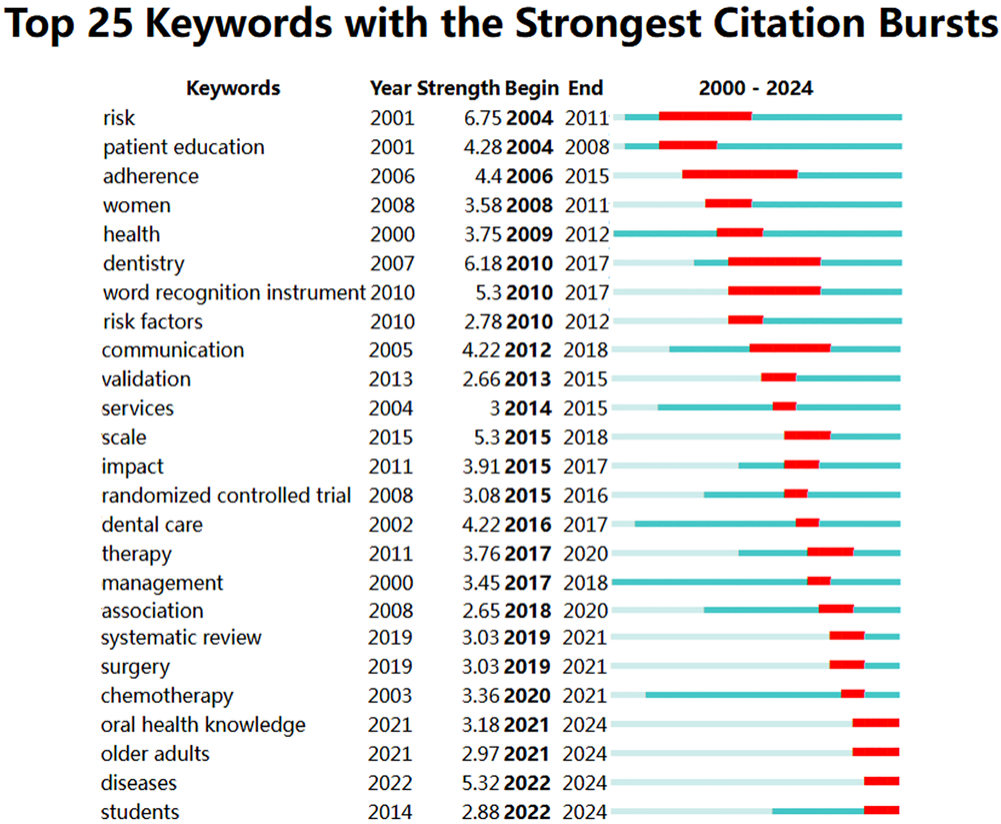
Burst detection analysis of keywords in studies on the impact of educational background on oral health from 2000 to 2024.

Figure 10 highlights the top 25 keywords with the strongest citation bursts. Based on the duration of burst and strength, the early stage (2000–2009) was characterized by research frontiers such as risk, patient education. The mid-stage (2010–2019) saw research focusing on keywords like adherence, dentistry, word recognition instrument, and communication. In recent years (2020–2024), research frontiers have shifted toward oral health knowledge, older adults, diseases, and students.

## 4. Discussion

This study employs bibliometric analysis to systematically investigate the current state, research hotspots, and developmental trends of studies examining the impact of educational background on oral health. Research in this field has experienced significant growth, particularly entering a phase of rapid development after 2020. The USA, the UK, Australia, China and Brazil have demonstrated prominent bibliometric performance in this domain, maintaining a dominant position. Health literacy, oral health, knowledge were the overarching research themes in this field. In the recent years, the research emphasis has shifted to more refined and early-preventive investigations.

In recent years, the international community’s growing focus on health equity has driven rapid advancements in research on education as a social determinant of health.^[22,23]^ Consequently, the significant increase in the number of studies may align with shifts in the global public health agenda. In this study, a notable surge in publications was observed during 2019 to 2020, coinciding with the outbreak of the COVID-19 pandemic. This global health crisis placed immense strain on healthcare systems and exacerbated the impact of social inequalities on oral health.^[24]^ The fear of the pandemic has exacerbated dental anxiety, particularly among individuals with lower educational levels, making it more difficult for them to seek dental visits compared to the period before the pandemic.^[25]^ Besides, according to the United Nations’ 2022 World Population Prospects report, both the number and proportion of the elderly population are continuously increasing.^[26]^ The aging process is accompanied by the exacerbation of education-related social inequalities,^[27]^ which have a detrimental impact on oral health. These factors may have contributed to an acceleration of the production of related research. Future studies are likely to delve further into how education, within the context of global health emergencies, can either alleviate or intensify health inequalities.

Although this research field continues to develop actively, most studies are concentrated in developed countries. An analysis of rankings across countries, institutions, authors, and journals reveals that, among developing countries, only China and Brazil exhibit significant research activity. Education, influenced by economic status, regional factors, and cultural contexts, displays notable disparities between developed and developing countries.^[28]^ The developed countries have well-established systematic education. However, the unequal distribution of educational resources in developing countries results in disadvantaged groups and individuals in remote areas having limited access to oral health knowledge and educational resources. This disparity contributes to a higher prevalence of untreated cases of dental caries and periodontal diseases.^[29]^ Therefore, fostering communication and collaboration between these regions and enhancing institutional partnerships are essential for further investigating the shared mechanisms through which education impacts oral health, ultimately contributing to the universal applicability of research findings.

Global burden of oral health issues remains severe, particularly among vulnerable groups like children and the elderly. Early childhood caries affected 600 million children worldwide.^[29]^ 353 million people worldwide suffer from edentulism, with more than 30% of people over 80 years old.^[30]^ The field of educational impact on oral health is predominantly focused on research related to oral health education, with the most attention being given to keywords such as health literacy. Through various educational interventions, individuals’ oral health literacy can be improved, which in turn improves patients’ adherence to medical instructions, self-management skills, and overall treatment outcomes,^[31]^ ultimately promoting overall oral health. The focus of oral health education and management varies depending on the target population. Early education is a critical intervention,^[32,33]^ as structured programs for children can substantially improve their disease prevention awareness and help establish lifelong healthy habits, while programs for adults focus on disseminating scientific oral care knowledge to reduce disease progression risks.^[34]^ Effective oral care for the elderly typically requires collaboration among healthcare providers, caregivers, and family members, all of whom need access to specialized oral health education.^[35]^

The temporal trend of the research demonstrates a progression from a broad to a more refined focus. Based on keyword clustering over time, the initial decade was a period of preliminary exploration, with a general engagement across various fields. As the research progressed into the mid-stage decade, the scope of the studies narrowed, with the overall research focus shifting towards adherence, dentistry, word cognition instruments, and communication. This indicates a gradual concentration on the more specific aspects of humanistic care in oral health. In recent years, the field has seen an explosion of research, primarily diving deeper into the finer details within the area, such as oral health knowledge, older adults, diseases, and students. It should be noted that a new cluster called “association” emerged in this phase, which, according to the cluster summary, focuses mainly on research concerning pregnant women, breastfeeding, and home-based health promotion. A longitudinal study spanning 25 years has shown that impaired oral health in children increases the likelihood of adverse dental experiences, which in turn can lead to long-term detrimental effects on oral health in adulthood.^[36]^ Multiple studies have demonstrated that providing oral health education interventions to parents of infants, and advancing the preventive time point for children’s oral health, can significantly improve the oral health status of infants.^[37–39]^ This is beneficial for enhancing the oral health of children and promoting lifelong oral well-being.^[40,41]^ Such educational approaches typically include verbal guidance, audiovisual presentations, and group discussions, with a focus on topics like infant oral health, the influence of maternal oral health on the child, and preventive measures.^[37,42,43]^ For instance, educating new mothers about infant oral health through video resources has been shown to significantly enhance their level of awareness.^[43]^

For future research on the impact of educational background on oral health, this study makes the following recommendations: Investigate how educational strategies can mitigate disparities in oral health inequalities during crises such as pandemics or the aging population process, especially focusing on addressing the challenges faced by vulnerable groups. Promote international collaboration, with a particular emphasis on low- and middle-income countries to address disparities in global oral health research. This includes exploring diverse educational models that reflect the socio-economic and cultural contexts of both developed and developing countries, especially in the area of oral health education. Explore subfields to develop more personalized, human-centered, and scalable education for underserved populations, with a particular focus on early-stage preventive research. These future directions can help bridge current knowledge gaps and provide evidence-based insights for policies and interventions aimed at improving oral health.

## 5. Limitations

This study, while comprehensive, acknowledges several limitations. First, the analysis was confined to articles indexed in the Web of Science Core Collection database. Although this database is widely recognized for its quality and breadth, it may not encompass all relevant publications, particularly those in non-English languages or those published in regional journals not indexed by WoS. This could result in underrepresentation of research from non-Western countries. Second, while bibliometric methods are effective for identifying trends and patterns, they cannot capture the nuanced content and quality of individual studies. The impact of a paper is not always accurately reflected by citation counts alone. Third, despite the carefully designed search strategy, the complexity and evolving nature of terminology in the fields of educational background and oral health may have led to the omission of some relevant articles. Furthermore, the time lag between research conduct and publication means that the latest developments in the field may not be fully reflected in this analysis. Lastly, although this study period extends to 2024, the indexing and citation processes require time, and recent data may be incomplete. Despite these limitations, the innovative aspect of this study lies in its ability to extract valuable patterns from vast amounts of literature, offering researchers an objective and comprehensive view of the field’s developmental dynamics, overall landscape, and research trends regarding the impact of education levels on oral health.^[44]^

## 6. Conclusion

This comprehensive bibliometric analysis offers valuable insights into the research landscape of the past 2 decades concerning the impact of educational background on oral health, highlighting the field’s active development and significant potential for growth. The findings underscore the necessity of strengthening international collaboration, conducting targeted research, and prioritizing vulnerable populations to effectively address oral health disparities associated with educational inequalities. These insights can inform policy-making and guide the development of more effective strategies to promote oral health equity across diverse populations.

## Acknowledgments

The authors would like to acknowledge that Gerhard Schmalz and Xianda Hu are co‑corresponding authors of this article.

## Author contributions

**Conceptualization:** Linxin Jiang.

**Data curation:** Linxin Jiang, Bincheng Liu.

**Formal analysis:** Bincheng Liu, Simin Li, Xianda Hu.

**Funding acquisition:** Linxin Jiang.

**Investigation:** Gerhard Schmalz.

**Methodology:** Shaohong Huang.

**Project administration:** Daniel R Reissmann, Xianda Hu.

**Resources:** Gerhard Schmalz, Xianda Hu.

**Software:** Shaohong Huang, Daniel R Reissmann.

**Supervision:** Daniel R Reissmann, Gerhard Schmalz, Xianda Hu.

**Visualization:** Linxin Jiang, Simin Li, Gerhard Schmalz.

**Writing –original draft:** Linxin Jiang.

**Writing –review & editing:** Daniel R Reissmann, Gerhard Schmalz, Xianda Hu.

## Supplementary Material


