# Supplementary material for: The evolution of the impact of educational background on oral health: A bibliometric and visualized analysis from 2000 to 2024
Source: Medicine (Baltimore). 2025 Aug 8;104(32):e43795. doi: 10.1097/MD.0000000000043795 (PMC12338237; doi:10.1097/MD.0000000000043795)
Supplement: Supplementary file 1 [file medi-104-e43795-s001.docx]

**APPENDIX TABLE 1 -** The classification information of each cluster

| **Cluster/ID** | **Size** | **Silhouette** | **Label (LSI)** | **Label (LLR)** | **Label (MI)** |
| --- | --- | --- | --- | --- | --- |
| Oral health education/0 | 59 | 0.781 | oral health education; multidimensional health locus; learning strategies; education; plaque control \| oral health; behavior; program; women; problem areas | oral health education (61.19, 1.0E-4); oral hygiene (21.64, 1.0E-4); oral health promotion (20.03, 1.0E-4); dental education (14.48, 0.001); school children (13.51, 0.001) | planning (0.53); problem areas (0.53); kerala (0.5 |
| Head and neck cancer/1 | 58 | 0.538 | oral health; oral health literacy; oral health knowledge; poor oral health; oral health disparities \| health literacy; dental caries; family relations; poor oral health; education | head and neck cancer (17.17, 1.0E-4); mendelian randomization (16.52, 1.0E-4); risk factors (14.66, 0.001); education attainment (11, 0.001); oropharyngeal cancer (11, 0.001) | hba(1c) (0.29); dietary supplements (0.29); public |
| Patient education/2 | 57 | 0.65 | patient education; pediatric patients; oral chemotherapy; systematic review; oral anticoagulants \| health literacy; clear writing; multidimensional health locus; group session; adjuvant chemotherapy | patient education (38.58, 1.0E-4); nursing (13.38, 0.001); oral health (12.86, 0.001); medication adherence (12.81, 0.001); oral health literacy (12.52, 0.001) | adjuvant chemotherapy (0.35); tomudex (0.35); afat |
| Tooth loss/3 | 52 | 0.769 | oral health; tooth loss; attachment loss; association; oslo citizens \| socioeconomic factors; public health; opioid reduction; prostitution; attitudes | tooth loss (24.75, 1.0E-4); epidemiology (15.73, 1.0E-4); socioeconomic factors (13.19, 0.001); pain (12.15, 0.001); attachment loss (11, 0.001) | energy (0.29); coffee consumption (0.29); chronic |
| Health literacy/4 | 50 | 0.876 | health literacy; intravenous bisphosphonate; comparative adherence; oral bisphosphonate; bladder cancer \| oral health; pregnant women; scoping review; patient compliance; community development | health literacy (29.64, 1.0E-4); oral health education (10.38, 0.005); comprehension (9.04, 0.005); pediatrics (8.87, 0.005); oral chemotherapy (7.04, 0.01) | doctor patient communication (0.55); interactive h |
| Oral health/5 | 49 | 0.851 | oral health; health education; community health workers; education methods; sleep disturbance \| dental caries; tooth injuries; dental care; sleep disturbance; stroke knowledge | oral health (59.75, 1.0E-4); health education (54.07, 1.0E-4); dental caries (40.11, 1.0E-4); early childhood caries (18.75, 1.0E-4); patient education (12.71, 0.001) | epidemiology. (0.82); special child (0.82); social |
| Internet/6 | 46 | 0.81 | health literacy; oral health-promoting behaviour; logistic regression analysis; patient compliance; education \| knowledge; dental care; patient education; communication; internet | internet (17.48, 1.0E-4); communication (12.78, 0.001); dental care (9.83, 0.005); dental health education (9.22, 0.005); population (9.22, 0.005) | general anesthesia (0.52); early childhood cary (0 |
| Care/7 | 46 | 0.764 | care; knowledge; association; adults; clinical attachment loss \| oral health; communication techniques; dentists; readability; clinical attachment loss | care (12.38, 0.001); diabetes mellitus (10.13, 0.005); blood glucose (9.55, 0.005); dental fear (9.55, 0.005); asthma (6.59, 0.05) | pre-exposure prophylaxis (0.45); oral exchange (0. |
| Oral health literacy/8 | 45 | 0.597 | oral health literacy; psychometric properties; cross-cultural adaptation; addisons disease; health professions education \| health literacy; oral health; addisons disease; health professions education; psychometric properties | oral health literacy (70.94, 1.0E-4); oral health status (21.61, 1.0E-4); validity (20.45, 1.0E-4); reliability (13.72, 0.001); instrument (8.96, 0.005) | acceptability (0.59); health literacy measurement |
| Association/9 | 44 | 0.777 | health literacy; pregnant women; cross-sectional study; breastfeeding assessment; instrument development \| oral health literacy; education; lifestyle-related risk factors; preschool-age children; human milk intake | association (15.62, 1.0E-4); dental cary (11.1, 0.001); oral health education (9.97, 0.005); exclusive breastfeeding (9.02, 0.005); preschool children (9.02, 0.005) | home-based health promotion (0.53); dentition (0.5 |
| Certified diabetes educators/10 | 15 | 0.898 | diabetes self-management training; certified diabetes educators; oral health education; glutamic acid; atrial fibrillation \| short message; group education; primary care; glutamic acid; atrial fibrillation | certified diabetes educators (19.27, 1.0E-4); diabetes self-management training (19.27, 1.0E-4); glutamic acid decarboxylase (9.6, 0.005); dementia (9.6, 0.005); glucose (9.6, 0.005) | glutamic acid decarboxylase (0.02); dementia (0.02 |
| Tobacco use cessation/11 | 9 | 0.978 | tobacco use cessation; smokeless tobacco; educational status; pregnancy outcome; chronic bronchitis \| periodontal treatment; observational data; causal estimates; tobacco use cessation; smokeless tobacco | tobacco use cessation (10.6, 0.005); neoplasms (10.6, 0.005); periodontal treatment (10.6, 0.005); pregnancy outcome (10.6, 0.005); morbidity (10.6, 0.005) | oral health (0.03); health literacy (0.02); tobacc |
